# Understanding what is visible in a mirror or through a window before and after updating the position of an object

**DOI:** 10.3389/fnhum.2014.00476

**Published:** 2014-06-27

**Authors:** Marco Bertamini

**Affiliations:** Department of Experimental Psychology, University of LiverpoolLiverpool, UK

**Keywords:** Venus effect, perspective, mirrors, spatial cognition, visual art

## Abstract

In the Venus effect observers assume that Venus is admiring her own reflection in the mirror (Bertamini et al., 2003a). However, since the observer sees her face in the mirror, Venus is actually looking at the reflection of the painter. This effect is general because it is not specific to paintings or to images of people. This study tests whether people have difficulties in estimating what is visible from a given viewpoint using a paper and pencil task. Participants (*N* = 80) judged what is visible in a scene that could include a mirror or an aperture. The object in the scene (a train) was already located in front of the mirror or behind the aperture, or the same object had to be imagined to move to that location. The hypothesis was that this extra step (spatial transformation) is always part of how people reason about mirrors because they have to imagine the location of the reflection based on the location of the physical object. If so, this manipulation would equate the difficulty of the mirror and of the aperture conditions. Results show that performance on the paper and pencil task was better than expected, probably because of the asymmetric nature of the object used. However, an additional cost in reasoning about mirrors was confirmed.

## Introduction

**Figure 1:**
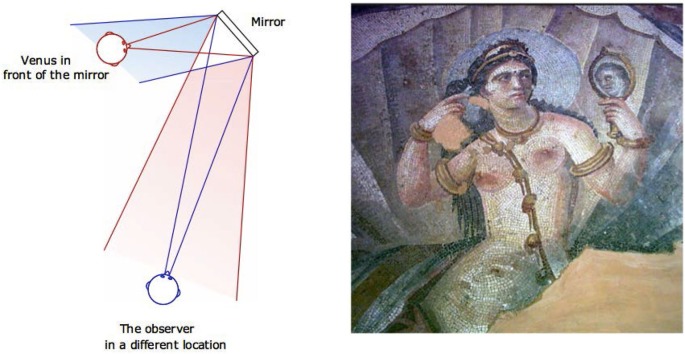
**The diagram shows that what Venus sees is different from what the observer sees**. For the observer to see Venus’s face, Venus should see the observer’s face. On the right is an early example of the toilette of Venus/Aphrodite. This mosaic (3rd century) found in the environs of Philippopolis is now in the Suweida Museum (Syria).

The Venus effect arises in situations where an observer looks at a scene in which another person is present together with a mirror, and the observer can see both the person and the person’s image reflected in the mirror. There is a tendency for the observer to say that the person in the scene is able to see a reflection of their own face, although this is not normally possible given the layout of the scene (Bertamini et al., 2003a). Figure 1 illustrates the geometry of what is visible from two different viewpoints. Next to the diagram there is an early example of a representation with Venus and a small mirror. This subject became very popular during the Renaissance, and there are many examples of the Venus effect from famous paintings. We can cite as one example the Venus with a mirror by Titian (*circa* 1555), and the later Rokeby Venus from Velasquez (*circa* 1647).

Artists have been fascinated by mirrors as much as everybody else. Melchoir-Bonnet (2002) and Pendergrast (2004) provide fascinating histories of mirrors across the centuries. It is possibly that recent work on mirror cognition can explain some of the mystery surrounding mirrors. In particular the Venus effect illustrates a surprising pattern in how scenes that include mirrors are described by most observers. Although the name Venus effect originates from an analysis of paintings, the effect itself is much more general. Bertamini et al. (2010) demonstrated the effect in photographs and in real life (i.e., a physical environment). They also tested the role of what is visible within the mirror. Surprisingly, having the face of the person visible in the mirror was not necessary for the effect. Most people tend to report that a person can see her own reflection when she appears near a mirror. The effect is related to another well-known effect: many participants believe that when approaching a flat mirror, one can see oneself before arriving in front of it. This has been referred to as the early error (Croucher et al., 2002; Bertamini et al., 2003b), and it appears to be stronger in adults than in children, suggesting a possible development of a mistaken strategy in adults (Bertamini and Wynne, 2010). Savardi et al. (2010) have proposed that this strategy is based on the combination of two beliefs: the belief that reflections *do the same* or the belief that they *do the opposite*.

Experiment 5 in Bertamini et al. (2010) is central for the purposes of this study and we will therefore describe it in more detail. Participants saw a top-down view of a room in which the observer’s location was indicated by a simple schematic drawing. The observer faced a wall with a mirror (represented by a line). Behind the observer there was another wall with nine nails (small lines). The task was to mark the nails that the observer could see in the reflection. The correct answer was that when the observer was to the left of the mirror she could see the nails to the right, when the observer was to the right of the mirror she could see the nails to the left, and when she was in front of the mirror she could see the nails in the middle of the wall. This is because different parts of the room were visible from the three different locations. However, there was a strong tendency to respond as if a similar central portion of the room was visible to a person located somewhere in the proximity of the mirror. This result shows a lack of sensitivity to the role of the viewpoint. Therefore, this could be the underlying cause of the Venus effect.

A surprising aspect of the Venus effect is that it suggests an inability of the observer to notice when another individual is looking at them using a mirror. This is in contrast with evidence that sensitivity to gaze direction in the human species is excellent, especially the ability to know whether another person is making eye contact (Gibson and Pick, 1963). It is also likely that the large visible sclera (white) in the human eye serves a role in communication through gaze (for a review, Emery, 2000).

Mirrors appear to have a special fascination for many people, and have been associated with power and magic. It is possible that one source of what is mysterious about mirrors is the fact that they create a virtual copy of the environment, and that the relationship between real and virtual is misleadingly simple. On the other hand there are also difficulties with understanding perspective that are not related to mirror reflection. It is possible that at the origin of the effect is something fundamental about how vision works.

One well-known example is the belief in extramission. Both children and adults believe that light travels from the eye to the object, and this is what makes vision possible (Winer et al., 2002). Developmental evidence is particularly interesting. Starting from the famous three-mountains experiment by Piaget and Inhelder (1967), researchers have studied children’s problems in perspective taking. Some basic knowledge, however, appears to be present in children by the age of 5 years. Flavell et al. (1981) found that children were able to appreciate that asymmetrical objects will look different to observers in different locations.

The results from Bertamini et al. (2010) suggest that the Venus effect is an example of a difficulty to understand the role of the viewpoint that affects pictures, photographs and real scene. However, one issue that is still unclear is how much this difficulty is linked to mirrors. The present study was designed to test observers’ sensitivity to what is visible in a mirror, and compare that to observers’ sensitivity to what is visible through a window.

## Experiment

We used a paper and pencil task to test participants’ ability to judge what is visible to an observer from a given viewpoint. In particular, very similar stimuli were presented and in one case a line in the scene was described as a mirror, and in the other case it was described as the glass pane of a window. The scene always included a train with nine carriages. Participants were asked which carriages would be visible to the observer. When the train is on the other side of a window a simple strategy becomes available, namely using the direct line of sight from the observer to the train. Therefore, in addition to a train visible on the other side of the window we added a condition in which the train had to travel to the final destination. Similarly, we also included a condition in which the train had to travel before arriving in front of the mirror. We reasoned that the difficulty in understanding what is visible in a reflection might be due to the extra step of imagining a transformation of the physical object into the virtual object. If so the condition in which the train had to be imagined to travel should make the window condition comparable to the mirror condition.

## Method

### Participants

Eighty students from the University of Liverpool took part in the experiment (age range: 18–21; 40 females). All participants had normal or corrected-to-normal vision. The experiment was approved by the Ethics Committee of the University of Liverpool and was conducted in accordance with the Declaration of Helsinki (2008).

### Design and procedure

**Figure 2:**
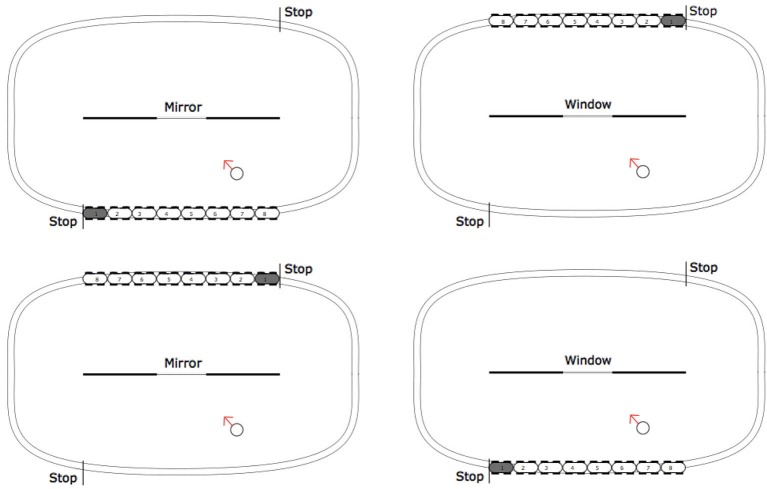
**Stimuli used in the four experimental conditions**. Top Left: Mirror reflection with movement of the train (MM). Bottom Left: Mirror reflection with static train (MS). Top Right: Window with movement of the train (WM). Bottom Right: Window with static train (WS).

The stimuli were simple diagrams printed on A4 paper. They depicted a top-down view of a train track. The images are shown in Figure 2. The four conditions are the results of a 2 × 2 design. In two of the conditions the central line was described as a “mirror”, and in the others as a “window”. In two conditions the train was static and the task was simply to say which carriages were visible. In the other two images the train was in a starting position, the task was to imagine the travel from the first stop sign until the second stop sign. After the end of the imagine travel participants had to report which carriages were visible (in the imagined situation). The correct answer for the mirror condition was the set 1, 2, and 3. The correct answer for the window condition was the set 6, 7, and 8.

Each participant was tested individually and only in one of the four conditions. They were given the relevant A4 paper diagram and an explanation of the map. In particular, they were told to imagine that they were the individual represented in the scene by the circle, and that the arrow was showing the direction in which they were looking. Afterwards they were asked the following question: “Which carriages would you see through the window [reflected in the mirror] from your position? Please circle them on the diagram”.

### Results and discussion

**Figure 3:**
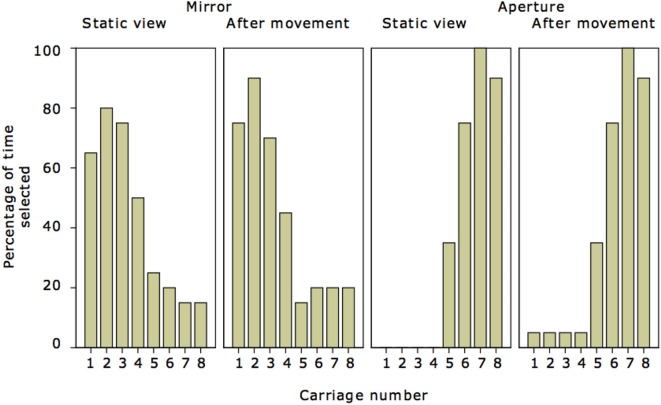
**For each of the four conditions the graphs show the number of times that each carriage was selected, as a percentage**. As there were 20 participants in each condition 100% means that that carriage was selected 20 times.

The results are illustrated in Figure 3. For the window conditions the correct carriages (6, 7, 8) were chosen, with a few additional responses for carriage 5. Overall performance was therefore very good. The differences between direct view and the imagine condition were minor.

For the mirror conditions the correct carriages (1, 2, 3) were chosen more than the others, but responses were more evenly distributed. Based on the results in Bertamini et al. (2010) we expected a more symmetrical distribution of responses. Overall, performance was therefore good, but not as good as in the window conditions. The asymmetrical distribution of responses is probably due to two important differences with respect to the diagram used in Bertamini et al. (2010). The first difference is that the train was an object with a clear asymmetry. Because we felt that we had to mark the engine as different from the rest of the carriages, the front of the train was gray (see Figure 2). The nails in Bertamini et al. (2010) instead were all identical. The second difference, perhaps even more important is that to indicate the direction of gaze we included in the diagram an arrow, pointing from the observer in the direction of the window/mirror. This was a highly salient feature of the diagram and indicated not only a direction but also a line of sight to some extent. Following this line and making it bounce off the mirror surface would provide a useful strategy for the participants to adopt.

Tables 1 and 2 summarize the responses in terms of participants who responded correctly and also in terms of the number of selected carriages. A chi square did not confirm any difference in overall number of participants who responded correctly or incorrectly (χ^2^ = 2.99, *p* = 0.083) in the mirror and window conditions, but a separate chi square confirmed a difference (χ^2^ = 14.56, *p* < 0.001) in terms of carriages. More incorrect carriages (49) were selected in the mirror condition, suggesting that this was a harder task.

**Table 1:** **Number of participants (out of 20) who selected the correct carriages in each condition**.

	**Exclusive**		**Non-exclusive**	
	**Static**	**Imagined motion**	**Static**	**Imagined motion**
Window	8	7	14	13
Mirror	4	4	9	11

*The exclusive criterion requires only the correct carriages to be chosen, the non-exclusive criterion accepts also cases in which additional carriages were selected*.

**Table 2:** **Number of correct carriages (out of 20 × 3 = 60) selected in each condition, and also number of incorrect carriages (out of 20 × 5 = 100) selected in each condition**.

	**Correct**		**Incorrect**	
	**Static**	**Imagined motion**	**Static**	**Imagined motion**
Window	53	53	7	11
Mirror	44	47	25	24

## Discussion

This study was based on previous work, and in particular a paper and pencil task used in Bertamini et al. (2010). It had emerged that there was a striking inability by most observers to understand that what is reflected in a mirror depends on the location of the observer. This error could explain the Venus effect in that people may not be able to appreciate what should be visible in a mirror and what is visible to different observers. As a consequence as long as they perceive a person near a small mirror they tend to guess that the person can see herself in it. It is, however, important to understand if the difficulty with reflection is actually specific to mirrors, or whether it is always difficult to know what is visible to different observers.

*Prima facie* one would expect mirrors to be more complex, because of the transformation that matches the virtual objects and the physical objects (Bertamini and Parks, 2005). However, with respect to the specific task of saying which objects are visible using a top down diagram one only has to imagine the objects to be transposed to the other side of the wall. In this study therefore we compared observers judgments of what would be reflected in a mirror and what would be visible though an aperture. In addition, we included a case in which the object had to be imagined as moving before arriving at a location, where it would be visible through the aperture. If the mirror difficulty is comparable to the window condition in which a transformation has to be imagined we would find a similar level of performance in these two conditions. The results did not support this hypothesis. The mental transformation did not significantly affect performance. Instead the only difference was between the aperture condition and the mirror condition, with the latter leading to more errors. The type of error was similar to that reported by Bertamini et al. (2010). Observers expected that more of the items in front of the mirror would be visible, despite the fact that the observer was located to the side. This bias, however, was much weaker than in the original study. In other words observers in this study performed better than we had anticipated. This is likely to be a consequence of the fact that a highly salient arrow was included in the diagram, providing visible information about the line of sight, and also because the object (a train) was asymmetrical.

The Venus effect is a striking illusion that applies to how observers interpret pictures, images, and scenes. It can therefore be used, consciously or unconsciously, by painters, movie directors and other visual artists. The origin is a difficulty in understanding the role of the viewpoint in scene interpretation, but mirrors provide a specific and unique problem and most observers fail to consider how the viewer’s location and the location of the mirror surface interact to determine what is visible.

## Conflict of interest statement

The author declares that the research was conducted in the absence of any commercial or financial relationships that could be construed as a potential conflict of interest.
